# Focused ion beam preparation of microbeams for *in situ* mechanical analysis of electroplated nanotwinned copper with probe type indenters

**DOI:** 10.1111/jmi.12868

**Published:** 2020-02-18

**Authors:** STUART ROBERTSON, SCOTT DOAK, FU‐LONG SUN, ZHI‐QUAN LIU, CHANGQING LIU, ZHAOXIA ZHOU

**Affiliations:** ^1^ Department of Materials Loughborough Materials Characterisation Centre, Loughborough University Loughborough U.K.; ^2^ Wolfson School of Mechanical, Electrical and Manufacture Engineering Loughborough University Loughborough LE11 3TU U.K.; ^3^ Institute of Metal Research Chinese Academy of Sciences Shenyang P.R. China; ^4^ Shenzhen Institute of Advanced Electronic Materials, Shenzhen Institutes of Advanced Technology Chinese Academy of Sciences Shenzhen P.R. China

**Keywords:** Chunk lift‐out, focused ion beam, *in situ*, micromechanical testing, nanotwinned copper, P‐FIB

## Abstract

A site‐specific xenon plasma focused ion beam preparation technique for microcantilever samples (1–20 µm width and 1:10 aspect ratio) is presented. The novelty of the methodology is the use of a chunk lift‐out onto a clean silicon wafer to facilitate easy access of a low‐cost probe type indenter which provides bending force measurement. The lift‐out method allows sufficient room for the indenter and a line of sight for the electron beam to enable displacement measurement. An electroplated nanotwinned copper (NTC) was cut to a 3 × 3 × 25 µm microbeam and *in situ* mechanically tested using the developed technique. It demonstrated measured values of Youngs modulus of 78.7 ± 11 GPa and flow stress of 0.80 ± 0.05 GPa, which is within the ranges reported in the literature.

**Lay Description:**

In this paper a site specific method is present for making particularly small mechanical tests samples, of the order of 100^th^ the size of a human hair. These small samples can then be used to determine the mechanical properties of the bulk material. Copper with a nano twinned grain structure is used as a test medium.

Ion milling was used to cut the sample to shape and a micro probe was used for mechanical testing. Ion milling can cut away very small volumes of material as it accelerates ions at the surface of the sample, atomically machining the sample. Micro probes are a cost‐effective small‐scale load measurement devices, however, they require a large area for accessing the sample. The indenter requirements are a problem when making you samples with ion milling as ion millers are best at making small cuts. Our aim was to design a cutting strategy which reduces the amount of cutting required while allowing samples to be fabricated anywhere on the sample.

We used a chunk lift out technique to remove a piece of material which is then welded to a wafer of silicon this gives sufficient space around the sample for ion milling and testing. The additional space allowed easy access for the probe. A 3 × 3 × 10 µm micro cantilever beam was cut out from copper, this beam was then bent. The force from bending and distance bent was measured and converted into Youngs modulus which is a measure of flexibility. The modulus value measured was comparable to the values reported in other papers.

## Introduction

The introduction of twins is a promising method for the strengthening of copper without reducing its ductility or electrical conductivity (Lu *et al*., 2004; Hasegawa *et al*., 2015; Cheng *et al*., 2017). [111] Orientated and nanotwinned copper (NTC) may be more capable of resisting electromigration and is promising for future high‐density and/or three‐dimensional integrated circuitry packaging (3D‐IC) (Chang *et al*., 2016; Sun *et al*., 2018a). Key to NTC's application to 3D‐IC is understanding its mechanical properties, thus far NTC has been found to be dependent on twin frequency, strain rate and crystal orientation (Lu *et al*., 2009; Ye *et al*., 2012; Kobler *et al*., 2015).

Micromechanical testing offers the ability to test small structures and features while providing well‐defined stress fields (Haque & Saif, 2003; Hemker & Sharpe, 2007; Pantano *et al*., 2012). From microbend testing flexural modulus, yield strength, flow stress, and fracture toughness can be determined (Di Maio & Roberts, 2005; Motz *et al*., 2005; Pantano *et al*., 2012).

Probe type indenters present a specific set of requirements for micromechanical sample preparation. The challenges are to provide space for indenter access and line of sight from the probe tip to the electron beam for displacement measurement. In the case of FIB prepared samples for probe type indenters, corner or edge sample preparation techniques are commonly used (Liu & Flewitt, 2013; Liu *et al*., 2014; Robertson *et al*., 2017). Due to the deficiencies of the edge/corner cutting techniques, a xenon plasma FIB (P‐FIB) chunk lift‐out based methodology is presented which can be used to test areas of interest from any location or multiple locations on a sample and allow batch mechanical testing.

## Experimental details

A FEI G4 CXe P‐FIB‐SEM (FEI, Oregon, USA) instrument at the University of Loughborough characterisation centre was used for the preparation of the microbeam chunk lift‐out. The P‐FB was equipped with an Easy Lift system and multichem gas injection system. A gallium FIB FEI Nova 600 Nanolab (FEI, Oregon, USA), equipped with a FMT 120, Kleindiek Nanotechnik probe (Kleindiek Nanotechnik, Reutlingen, German) was used to make the force measurements. The experiments were performed through the following procedures. A summary of the steps required is shown in Table 1.

**Table 1:** Summary table of steps required for microcantilever preparation including accelerating voltage, current and sample tilt (currents may vary dependent on the sputter rate of the material used)

Step	Process and patterning function	Accelerating voltage	Current	Sample tilt
**Step 1**	Pt deposition	16 keV	20 nA	52°
**Step 2**	Sample side cutting (regular cross‐section)	30 keV	2.5 µA	52°
**Step 3**	Under cutting (box milling)	30 keV	60 nA	0°
**Step 4.1**	Probe attachment (Pt deposition)	30 keV	0.1 nA	0°
**Step 4.2**	Chunk retaining cut (box milling)	30 keV	4 nA	0°
**Step 5**	Silicon cut to shape	30 keV	2.5 µA	52°
**Step 6.1**	Cut cantilever to shape (cleaning cross‐section)	30 keV	2.5 µA–15 nA	52° ± 1°
**Step 6.2**	Under cutting the cantilever (cleaning cross‐section)	30 keV	2.5 µA–15 nA	−38°
**Step 7**	Cut holding position (box milling)	30 keV	2.5 µA	0°
**Step 8**	Repeat steps 4.1–4.2 to move the sample to the holding position			
**Step 8.1**	Pt weld sample to the Si (Pt deposition)	16 keV	3 nA	52°

**Figure 1:**
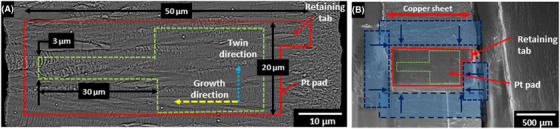
(A) The dimensions of the deposited Pt pad and the dimensions of the microbeam produced, the yellow arrow denotes the growth direction, while, the blue arrow denotes the twinning direction. (B) The ‘G’ shaped location of the cleaning cross section perimeter cuts.

Step 1: At 52° a pad platinum/carbon composite was deposited at 16 keV with a current density of 30 pA µm^–2^ to a thickness of 3 µm. The platinum carbon deposit is used to reduce damage and improve cutting performance. The area of the pad could be scaled depending on the microbeam required, the pad width should be 5–7 times the required width of the beam and pad length is deposited at ≈1.5 times the required length of the microbeam (see Fig. 1A).

Step 2: A ‘G’ shaped cut was made around the Pt pad consisting of cross sections leaving a tab of material linking the sample to the substrate. Cuts were made at 30 keV and 2.5 µA. Each cut was 20 µm width and 100 µm depth. A schematic representation of this cutting procedure can be seen in Figure 1(B).

**Figure 2:**
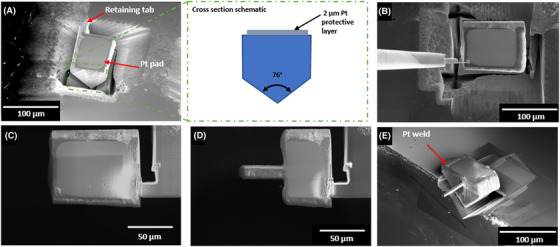
(A) The prepared chunk lift‐out with dimensions (70 × 100 × 60 µm), a cross‐section of the chunk is shown schematically. (B) An electron image of the removal of chunk from the bulk using the EasyLift system. (C) The chunk lift‐out mounted on a silicon substrate for machining. (D) Profile machining of the chunk lift‐out to dimension and located in a docking test position. (E) The completed microcantilever.

Step 3: At 0° tilt, the ion column is incident at 38° to the sample, depending on the size of the chunk required, and propensity for the material to redeposit cutting from both or one side can be conducted to free the chunk. Undercuts were made along the long edges of the chunk with box type cuts of width 5 µm and depth of 100 µm. The sample was rotated, and undercuts repeated. This process resulted in a free‐standing chunk with an ‘inverted house’ cross section (see Fig. 2A).

Step 4: The sample was then Pt welded to the EasyLift probe and the tab connecting the chunk to the bulk cut (see Fig. 2B for detail).

Step 5: A freshly fractured piece of silicon is then used as a mounting point for the sample. At the corner of the silicon, a post is machined to receive the sample to allow for undercutting of the microbeam. The chunk can then be docked on the post and Pt welded in a manner similar to that which is used for TEM lift outs.

Step 6: Sample machining to final dimension was completed using the cleaning cross‐section at 52° at 30 keV and 2.5 µA (as shown in Fig. 2D). Once the beam is thinned to 1.5 times the required thickness the sample can be undercut. Tilting the stage to ‐38° allows the ion beam to be parallel to the microbeam, the cantilever can then be machined to the final dimension with concurrently lower ion beam currents, in this case from 2.5 µA to 15 nA. To ensure parallelism of the beam faces it is advised that final thinning be conducted at ±1° to the milling angle.

Step 7: To ensure a sufficient bond between the chunk and Si substrate a ‘holding’ point was milled using a cleaning cross section cut at 0° tilt 30 keV 2.5 µA. The cantilever was placed in the holding station in a similar manner to step 5. Thick (3 µm) Platinum welds were used to ensure the sample was fixed to the silicon substrate. The easy lift probe was cut from the sample. The final machined beam is shown in Figure 2(E).

Bend testing was completed inside the Ga+ FIB due to the additional pole piece working distance. The cantilever and probe were aligned. The Y stage of the microscope was scripted to index in 0.1 µm steps towards the indenter bending the cantilever. A video and time stamped images were recorded to link force and displacement.

Following microbend testing an in‐plane transmission electron microscope lift‐out was prepared from the deformed region including base of the cantilever.

## Results and discussion

**Figure 3:**
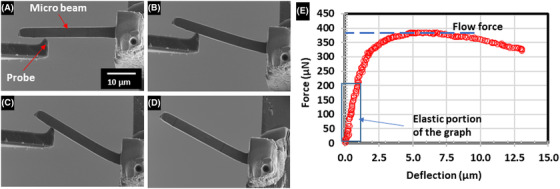
(A)–(C) The progress of deformation during microbend testing. (D) The plastically deformed copper nanotwinned cantilever. (E) The force deflection plot from the bend test.

The microcantilever fabricated had a width of 3.45 ± 0.05 µm, height of 3.72 ± 0.15 µm and length of 25.0 µm. The microbeam was found to significantly plastically deform (see Figs. 3A–D).

No deflection of the base material relative to the silicon substrate was measured indicating the Pt welds held the sample sufficiently. Based on the images and force‐time data recorded a force‐displacement plot was produced (see Fig. 3E). Standard square cross‐section bending equations were used to determine modulus based on the elastic portion of the force deflection graph 17. The Young's modulus of the copper nanotwins was found to be 78.7 ± 11 GPa (error calculated based on cross section variation). The Young's modulus of copper at the macro scale is widely accepted as 117 GPa; however, depending on the crystallographic orientation of individual grains, the copper's modulus can vary from 59–77 GPa in the [001] direction up to 160–202 GPa in the [11 ®1] direction (Armstrong *et al*., 2009; Ye *et al*., 2012).

**Figure 4:**
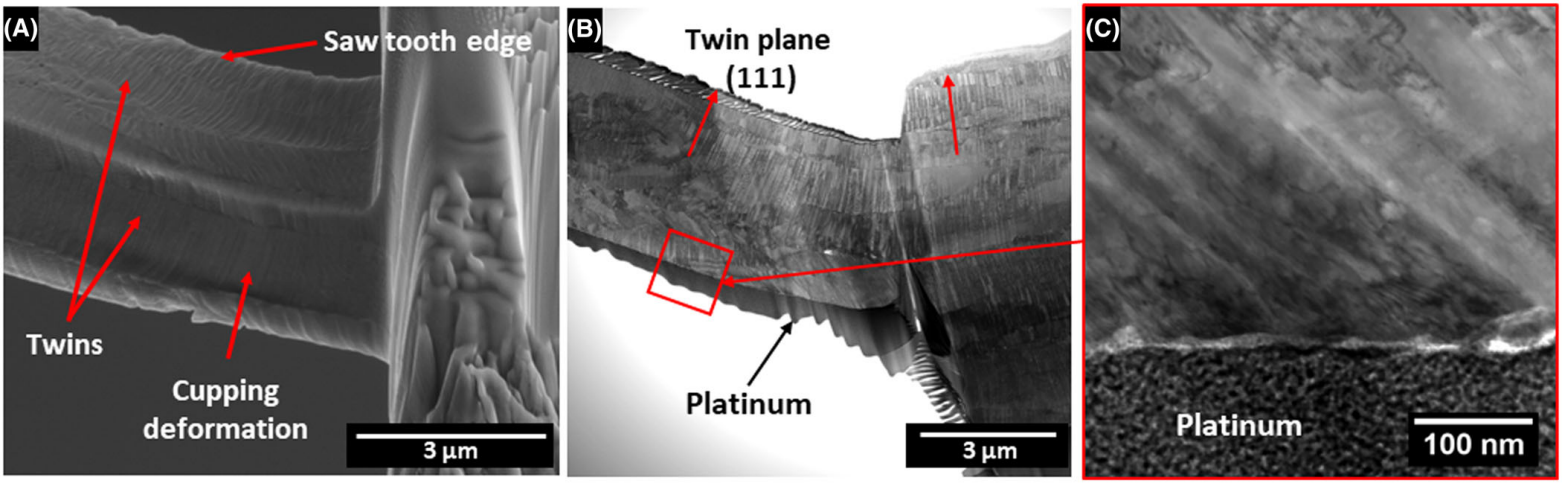
(A) An electron beam image recorded at 45° tilt showing the cupping deformation of the tensile face of the microbeam. (B) Cross section bright field scanning transmission electron micrograph of the deformed copper cantilever. (C) A magnified section from image (B) of the tensile ion beam machined face, no clear modification of the surface of the cantilever is observable.

The twins in the sample were on the (111) plane with a frequency ranging from several nm to 100 nm with a mean frequency of 22 nm (see Fig. 4B) (Sun *et al*., 2018a, Sun *et al*., 2018b). The bend test was conducted parallel to the (111) plane, and the cantilever was polycrystalline as such no single value for the crystal orientation tested can be determined. Following testing the tensile face exhibited cupping deformation (see Fig. 4A). This cupping deformation demonstrates the high ductility of the copper nanotwin material. The flow stress of the copper microbeam was calculated to be 0.80 ± 0.06 GPa based on the methodology presented by Motz *et al*. (2005). The estimated flow stress of the nanotwinned copper is higher than those reported for equiaxed copper microbeams of a comparable size (3.5 × 3.5 µm) in the literature, being 0.334 GPa 13. This increase in flow stress can be attributed to the strengthening effects of the twins in the sample blocking dislocation movement. STEM images from the ion beam milled tensile face of the cantilever do not show any significant modification, or amorphisation of the surface (Fig. 4D). Thus, it is expected that any modification in mechanical properties through FIB machining small when compared to the errors from SEM deflection measurements and force measurements. Motz *et al*. reported 0.5–1 GPa hardness increases to the first 300 nm of gallium ion implanted copper, such changes were deemed negligible when measuring >1 µm^2^ cantilevers (Motz *et al*., 2005). Furthermore, xenon ion milling has been shown to produce significantly less amorphisation when compared to gallium ion milling (Kelley *et al*., 2013).

## Conclusion

An efficient site‐specific chunk lift‐out methodology has been presented and applied to copper nanotwins, and a 3 × 3 × 25 µm beam cut parallel to the growth direction of the electroplated nanotwinned copper was produced. The microbeam was bend tested, the flexural modulus was found to be 78.7 ± 11 GPa, and the flow stress was 0.80 ± 0.06 GPa. The chunk lift‐out methodology was ideal for site specific micromechanical testing with a probe type indenter.

## References

[jmi12868-bib-0001] Armstrong, D.E.J. , Wilkinson, A.J. & Roberts, S.G. (2009) Measuring anisotropy in Young's modulus of copper using microcantilever testing. J. Mater. Res. 24(11), 3268–3276. 10.1557/jmr.2009.0396

[jmi12868-bib-0002] Chang, L. , Chen, C. , Hu, D. , Tain, R. & Chen, Y.H. (2016) Fabrication and characterization of electroplated nanotwinned‐copper films on polymer substrates. (IMPACT‐IAAC 2016). 198–200.

[jmi12868-bib-0003] Cheng, G. , Li, H. , Xu, G. , Gai, W. & Luo, L. (2017) In situ observation of nanotwins formation through twin terrace growth in pulse electrodeposited Cu films. Sci. Rep. 7(1), 1–10. 10.1038/s41598-017-10096-5 28963542 PMC5622094

[jmi12868-bib-0004] Di Maio, D. & Roberts, S.G. (2005) Measuring fracture toughness of coatings using focused‐ion‐beam‐machined microbeams. J. Mater. Res. 20(02), 299–302. 10.1557/JMR.2005.0048

[jmi12868-bib-0005] Haque, M.A. & Saif, MTA. (2003) A review of MEMS‐based miroscale and nanoscale tensile and bending testing. Exp. Mech. 43(3), 248–255. 10.1007/BF02410523

[jmi12868-bib-0006] Hasegawa, M. , Mieszala, M. , Zhang, Y. , Erni, R. , Michler, J. & Philippe, L. (2015) Orientation‐controlled nanotwinned copper prepared by electrodeposition. Electrochim. Acta 178, 458–467. 10.1016/j.electacta.2015.08.022

[jmi12868-bib-0007] Hemker, K.J. & Sharpe, WN. (2007) Microscale characterization of mechanical properties. Annu. Rev. Mater. Res. 37(1), 93–126. 10.1146/annurev.matsci.36.062705.134551

[jmi12868-bib-0008] Kelley, R. , Song, K. , Leer Van, B. , Wall, D. & Kwakman, L. (2013) Xe+ FIB milling and measurement of amorphous silicon damage. Microsc. Microanal. 19(Suppl 2), 862–863. 10.1017/S1431927613006302

[jmi12868-bib-0009] Kobler, A. , Hodge, A.M. , Hahn, H. & Kübel, C. (2015) Orientation dependent fracture behavior of nanotwinned copper. Appl. Phys. Lett. 106(26), 1–6. 10.1063/1.4923398

[jmi12868-bib-0010] Liu, D. & Flewitt, PEJ. (2013) The measurement of mechanical properties of thermal barrier coatings by micro‐cantilever tests. Key Eng. Mater. 525–526, 13–16. 10.4028/www.scientific.net/KEM.525-526.13

[jmi12868-bib-0011] Liu, D. , Heard, P.J. , Nakhodchi, S. & Flewitt, P.E.J. (2014) Small‐scale approaches to evaluate the mechanical properties of quasi‐brittle reactor core graphite. ASTM Int. 84–104. 10.1520/STP15782013012 7

[jmi12868-bib-0012] Lu, L. , Chen, X. , Huang, X. & Lu, K. (2009) Revealing the maximum strength in nanotwinned copper. Science 323(January), 607–610. 19179523 10.1126/science.1167641

[jmi12868-bib-0013] Lu, L. , Shen, Y. , Chen, X. , Qian, L. & Lu, K. (2004) Ultrahigh strength and high electrical conductivity in copper. Science 304(5669), 422–426. 10.1126/science.1092905 15031435

[jmi12868-bib-0014] Motz, C. , Schöberl, T. & Pippan, R. (2005) Mechanical properties of micro‐sized copper bending beams machined by the focused ion beam technique. Acta Mater. 53(15), 4269–4279. 10.1016/j.actamat.2005.05.036

[jmi12868-bib-0015] Pantano, M.F. , Espinosa, H.D. & Pagnotta, L. (2012) Mechanical characterization of materials at small length scales. J. Mech. Sci. Technol. 26(2), 545–561. 10.1007/s12206-011-1214-1

[jmi12868-bib-0016] Robertson, S. , Doak, S.S. , Zhou, Z. & Wu, H. (2017) In‐situ micro bend testing of SiC and the effects of Ga+ion damage. J. Phys. Conf. Ser. 902(1), 012002 10.1088/1742-6596/902/1/012002

[jmi12868-bib-0017] Sun, F.L. , Gao, L.Y. , Liu, Z.Q. , Zhang, H. , Sugahara, T. , Nagao, S. & Suganuma, K. (2018a) Electrodeposition and growth mechanism of preferentially orientated nanotwinned Cu on silicon wafer substrate. J. Mater. Sci. Technol. 34(10), 1885–1890. 10.1016/j.jmst.2018.01.016

[jmi12868-bib-0019] Sun, F.L. , Liu, Z.Q. , Li, C.F. , Zhu, Q.S. , Zhang, H. & Suganuma, K. (2018b) Bottom‐up electrodeposition of large‐scale nanotwinned copper within 3D through silicon via. Mater. 11(2), 319 10.3390/ma11020319 PMC5849016 29473865

[jmi12868-bib-0018] Ye, J.C. , Wang, Y.M. , Barbee, T.W. & Hamza, A.V. (2012) Orientation‐dependent hardness and strain rate sensitivity in nanotwin copper. Appl. Phys. Lett. 100(26), 261912 10.1063/1.4731242

